# Perceptions about future health trajectories among women at risk for developing cardiometabolic disease: A qualitative study

**DOI:** 10.21203/rs.3.rs-3386180/v1

**Published:** 2023-10-26

**Authors:** Jacqueline Kent-Marvick, Stephanie Lynn St. Clair, Alycia A. Bristol, Bryan Gibson, Sara E. Simonsen

**Affiliations:** University of Utah; University of Utah; University of Utah; University of Utah; University of Utah

**Keywords:** Cardiometabolic disease, Type 2 diabetes mellitus, Gestational diabetes mellitus, Hypertensive disorder of pregnancy, Prediabetes, Obesity, Women’s health, Lifestyle change, Health promotion, Qualitative research

## Abstract

**Background:**

The reproductive years provide a window into future risk for Type 2 Diabetes (T2DM); women’s risk is seven to 10 times higher after gestational diabetes (GDM) and two to four times higher after a hypertensive disorder of pregnancy (HDP). Targeting reproductive-aged women at high risk for T2DM could reduce future T2DM incidence. However, little is known about such women’s diabetes risk perceptions, or their knowledge or barriers/motivators of lifestyle change–information essential to understanding how to engage these at-risk women in tailored prevention programs promoting long-term health. This study’s aims include: among reproductive-aged women at high risk for T2DM, what is/are 1) personal health-risk awareness, 2) lifestyle-change interest, and 3) barriers/motivators of participation in lifestyle-change programs?

**Methods:**

Women aged 18 to 48 were eligible if they had one of the following health risks: 1) GDM or HDP during pregnancy, 2) prediabetes diagnosis, or 3) BMI classified as obese. Three Zoom focus groups, organized by risk group, were conducted with a total of 20 participants. Qualitative content and thematic analysis were used for the focus group transcriptions.

**Results:**

Women’s personal health-risk awareness was limited and generalized (e.g., being overweight might lead to other risks) and rarely reflected awareness connected to their personal health history (e.g., GDM increases their lifetime risk of T2DM). Participants reported that healthcare providers did not adequately follow or address their health risks. All women expressed interest in making healthy lifestyle changes, including engagement in formal programs, but they shared multiple barriers to healthy behavior change related to being “busy moms.” Women emphasized the need for social support and realistic solutions that accounted for the dynamics of motherhood and family life. Common motivators included the desire to maintain health for their families and to set a good example for their children.

**Conclusions:**

Participants lacked knowledge and were eager for information. Healthcare improvement opportunities include better coordination of care between primary and specialty-care providers, and more frequent communication and education on diabetes-related health risks and long-term health. Formal lifestyle programs should tailor content by providing multiple formats and flexibility of scheduling while leveraging peer support for sustained engagement.

## Background

More than one in five reproductive-aged women (i.e., aged 18–44) have prediabetes,^1,2^ a major risk factor for Type 2 Diabetes (T2DM),^3^ infertility,^4^ miscarriage,^4^ and adverse metabolic outcomes in their children.^5^ Obstetrical issues during pregnancy also impact a woman’s future risk for T2DM: her risk is seven to 10 times higher if she has had gestational diabetes (GDM)^6, 7^ and two to four times higher if she has had a hypertensive disorder of pregnancy (HDP).^8, 9^ Identifying and targeting reproductive-aged women at high risk for T2DM provides an opportunity for timely implementation of prevention strategies earlier in their life course and at the earliest stages of their children’s development.^10^ However, little is known about such women’s diabetes risk perceptions, or about their knowledge, beliefs, or hindrances and motivations related to lifestyle change.^10^ This information is needed to understand how to engage these at-risk women in prevention programs, effectively tailor motivational interventions, and prevent morbidity and mortality.

Engagement in formal lifestyle-change programs can impact long-term health trajectories.^11^ One such example is the National Diabetes Prevention Program (DPP), a lifestyle-change program sponsored by the Centers for Disease Control and Prevention.^11, 12^ This year-long healthy-lifestyle program effectively prevents T2DM, with a 58% reduction in diabetes incidence at three years. However, DPP enrollment rates are extremely low among reproductive-aged women^13^ despite the fact that DPP participation and related weight loss may result in a substantial reduction in their T2DM risk as they age,^11, 14^ and may also have a positive impact on their pregnancy outcomes and the health of their children.^15^ Thus, there is a critical need to understand more about the perspectives of reproductive-aged women at high risk for diabetes in order to effectively inform them, and to motivate them to engage in diabetes-prevention activities.

To better understand the experiences and health-risk perceptions of reproductive-aged women, there is a need for qualitative data from individuals who 1) have had a complication during pregnancy (i.e., GDM, HDP), 2) have already been diagnosed with prediabetes, or 3) are individuals with obesity.^16^ Such knowledge is needed to learn how women who are at risk for T2DM may be encouraged to participate in programs designed to improve their health during their childbearing years. Therefore, the aim of this study was to use focus groups with reproductive-aged women at high risk for T2DM in order to learn about 1) their awareness of personal health risk, 2) their interest in lifestyle changes, including participation in formal lifestyle-change programs such as the DPP and 3) barriers and motivators to participation in a formal lifestyle-change program. Additionally, focus group questions addressed participant perspectives about the optimal timing for risk notification (e.g., before pregnancy, between pregnancies, at an annual exam, during postpartum care), and the preferred methods for notification (e.g., conversation with healthcare provider, email or text message from health system, contact from a DPP coach).

## Methods

### Participants and Setting

Individuals were considered eligible for the study if they were a person who had given birth (referred to as women in this report), were aged 18 to 48 and had one of the following three health risks: 1) a complication during pregnancy (i.e., GDM, HDP), 2) a diagnosis of prediabetes, or 3) a BMI classified as obese.

Focus group participants were recruited by the University of Utah’s Center for Clinical and Translational Science Community Collaboration & Engagement Team (CCET), using fliers sent to eligible individuals within the CCET’s network of contacts. The CCET supports community-engaged research that addresses investigator- and community-identified priorities with the goal of promoting community health needs. Three focus groups, organized by risk group, were conducted online via Zoom in collaboration with the CCET. The format of focus groups, rather than individual interviews, was selected in order to facilitate a broad understanding of women’s knowledge and feelings about the topic. Specifically, the focus group setting was selected to promote social interactions and conversation so that key themes relating to the topic could be identified.^17^

### Qualitative Design

A qualitative descriptive approach was used to facilitate an understanding of women’s personal health risk, interest in formal lifestyle-change engagement, and barriers and motivators to lifestyle change. Following qualitative-descriptive methodology, the aim was to stay close to the data, using language that closely reflected the verbiage used by participants.

Data collection included audio-recording the one-hour-long focus group discussions, which took place between February and April of 2022. Questions were provided to participants prior to the Zoom meetings and were discussed during the focus group engagement sessions. These questions were:

1. What do you think/know about the relationship between [prediabetes, GDM, hypertensive disorders of pregnancy, obesity] and your risk for diabetes and heart disease, reproductive health outcomes such as fertility or complications in future pregnancies, and the health of your current or future children and family?
2. What is your experience talking about these risks with doctors or other healthcare providers?
3. How would you want to be notified about these risks?
4. What barriers prevent you from making lifestyle changes? What barriers prevent you from participating in a lifestyle-change or weight-loss program?
5. What would encourage you to make lifestyle changes? What would encourage you to participate in a lifestyle-change program or weight-loss program? How should women be notified about these programs?

Prompts were used by the focus group interviewer in order to encourage participants to provide additional details about their perspectives and experiences. Additionally, the interviewer noted commonalities and differences between participant perspectives and experiences in order to encourage additional participant insight regarding these similarities and divergences. Audio recordings of the focus groups were transcribed by a study research assistant and verified for linguistic accuracy by additional members of the research team.

### Analysis

Qualitative content and thematic analysis were used^18, 19^ to examine the data, and thematic saturation^20–23^ was reached during analysis of the focus group discussions. Deductive codes were developed based on concepts related to the research question (e.g., barriers and motivators) and interview question content. Deductive codes were used during the initial coding of the first transcript. A codebook was developed with identified definitions and was used to code the remaining transcripts. Additionally, inductive codes emerged during the coding process, representing novel insights shared by participants, and these were added to the codebook. After coding was completed, codes were organized into categories and themes. Authors A.A.B., J.K.M., and S.S. engaged in the initial coding of the transcripts. A fourth member of the research team (S.S.C.) performed an audit of the group’s notes and coding to assess for consistency and accuracy of coding. Disagreements regarding coding were discussed until consensus was reached among the group.

To address rigor, trustworthiness criteria outlined by Lincoln and Guba (1985) were used,^24^ including weekly coding meetings with authors (A.A.B, J.K.M., S.S., S.S.C.), during which the authors discussed nuances in the data to ensure consistency and reach consensus in the application of codes. The authors compared observations of coding across transcripts to assess similarities and differences between participant experiences not only as women who had given birth per se, but also as women with differences in health risks. A group memo document was maintained and reviewed during weekly meetings.

### Ethical Considerations

Prior to the study’s initiation, the study received a waiver-of-consent documentation from the University of Utah Institutional Review Board, due to the low-risk nature of the focus group sessions. Participants did receive a letter containing information about the study’s purpose and potential risks associated with participation. The voluntary nature of participation was also discussed with participants as a group at the beginning of each session. Participants received a $75 gift card in gratitude for their time and their contribution to the study.

## Results

Focus group participants were women who were mothers (n = 20) and who 1) had a complication during pregnancy [i.e., GDM, HDP; (n = 7)]; 2) had been diagnosed with prediabetes (n = 8); or 3) were individuals with obesity (n = 5). Participants were predominantly white (75%), non-Hispanic (85%), married or living with a partner (75%), and had a bachelor’s degree or higher (75%). The majority (60%) had no prior experience with a formal lifestyle-change program. Table 1 outlines the demographic characteristics of the sample.

In response to the focus group questions, women across the risk groups shared similar experiences with their healthcare providers, personal knowledge of their current and long-term risk, and ability to engage in healthy-lifestyle behaviors. The main themes identified through analysis were 1) *Women’s knowledge and understanding of their health risk,* 2) *improving healthcare for mothers at risk for cardiometabolic disease,* and 3) *Healthy lifestyle-change barriers and motivators.*

### Theme 1: Women’s Knowledge and Understanding of Their Health Risk

Women’s personal health-risk awareness was often only a general awareness, such as that extra body weight might lead to other health risks. Women often explained their risk awareness through anecdotal experiences, such as witnessing family members’ struggles with health issues. For example, one woman shared how her understanding of diabetes was shaped by her father’s health experience with Type 1 Diabetes:

The majority of my knowledge, to be honest, has to do with just personal experience. … with what my dad went through with Type 1 Diabetes. He actually passed away at the age of 54 from complications to his diabetes. He had early onset dementia that started at 52, and ultimately passed away from renal failure of the kidneys. … He was scheduled for dialysis, but hadn’t started it yet, when he passed away. And also ended up with some heart complications, and a couple of mini strokes. … neuropathy, the whole … it was a pretty bad situation for him. So, I feel like, I’ve seen kind of some of the worst that can happen. Or … really the worst that can happen from it. (Participant #6; prediabetes group; white, non-Hispanic)

This participant expressed her view that these experiences subsequently led her to prioritize diabetes prevention for herself. Another woman learned the full weight of the potential implications of her GDM while applying for health insurance after the birth of her child:

After my first pregnancy, I applied for life insurance. You know, just a new baby–life insurance. And I found it very interesting that although I had been diagnosed with prediabetes, the fact that I had had gestational diabetes … put me higher on the life-insurance risk category than even the prediabetes. The gestational diabetes was considered by the actuaries a higher risk. … That was kind of sobering to just realize that fact. And I know that the likelihood of developing Type 2 Diabetes is much, much higher because of my experience with gestational diabetes. (Participant #5; obesity group; white, Hispanic/Latino)

Additionally, women expressed interest in addressing their knowledge gaps and learning about how their personal health risks might inform future health outcomes. One woman with an obese BMI was unaware that her challenges with fertility and becoming pregnant could be connected to her personal health profile:

When I read the question … “Do you know about the relationship between these health conditions and health risks?” For example, “fertility and complications in future pregnancies,” … I was like, Oh! Maybe that is going to be something that I am going to have to take into consideration with future pregnancies if I am so fortunate to have, but my other thought was, I am being seen for infertility and I was for my little guy, too. We went and did IUI in order for me to get pregnant. … My husband and I are both unexplained infertility and so now I guess I am wondering if, like, maybe that has anything to do with it. I don’t know. And so, I guess just more than anything, I don’t have a lot to contribute as far as what I know, but it really just makes me like want the information. (Participant #3; pregnancy-complications group; white and Asian, non-Hispanic)

Expression of this type of wish for additional education was a common comment among the focus group discussions, and the expressed need for education was an issue that connected to the next theme, *improving healthcare for mothers at risk for cardiometabolic disease.*

### Theme 2: Improving Healthcare for Mothers at Risk for Cardiometabolic Disease

Broadly, participants frequently reported their belief that healthcare providers did not adequately follow and address their health risks. Additionally, participants made numerous comments about and provided suggestions for how reproductive women’s healthcare could be improved in order to address their long-term health. Two subthemes were created to explicate these findings: 1) *Healthcare inadequacies,* and 2) *Suggestions for healthcare improvement.*

#### Healthcare inadequacies

Across risk groups, the focus group participants detailed an overall description of their healthcare (i.e., healthcare that included their OBGYN and other primary providers) as lacking key qualities essential to their long-term health. Women attributed their lack of risk-related knowledge to care that was often perceived as dismissive and characterized by poor communication and education about their risk factors. For example, one woman, who was diagnosed with prediabetes by her primary-care provider, felt it necessary to obtain specialist care in order to address her new diagnosis. She stated that after her primary provider delivered the diagnosis, the doctor

Kind of left it at that. I remember they talked about, like, it was reversible. But they didn’t really give me any strategies. And it’s been my experience with managing it, I had to seek out a specialist on my own. And luckily, I was able to find a specialist who … he sits down with you for 30 to 60 minutes in an appointment. Whereas I feel like when I go to the primary-care physician or most doctors at most clinics, they only see you for five to 10 minutes. (Participant #2; prediabetes group; white, non-Hispanic)

In addition to perceiving their care as often dismissive and lacking the thoroughness they desired, women also believed this type of care put them at increased risk for poorer long-term health outcomes: “I felt like they made it more about just statistics, you’re statistically overweight that’s great and they just kind of glanced over me as a person. (laugh) Um, and which put me more at risk.” (Participant #7; pregnancy-complications group; white, non-Hispanic)

The issue of weight was raised frequently in relation to healthcare and ongoing health issues. Participants believed providers should address weight with sensitivity and not oversimplify weight’s relationship to health. Several participants stated that providers minimized ongoing health issues due to weight bias, putting them at risk for poor outcomes. Additionally, women said providers should understand that discussing weight and weight gain can be stressful and addressing weight or obesity should be a collaborative process, where providers work to connect women with the resources they need to begin to make healthy-lifestyle changes (e.g., a referral to a nutritionist). One woman described the complexity of addressing weight sensitively and in the context of her overall health:

I think that it can be very tricky because so often, it is related to weight. And weight loss. And I think how the providers approach that subject can be tricky, because … you can feel a little bit of shame about it, like, “oh, just 15 pounds, you know, could make such a difference in my health journey.” … Just in the hospital, a couple days ago, a doctor said, “well just stay skinny, and that’s all you have to do.” And while that is helpful, it’s not at the same time. … It’s complicated. There’s a lot of factors and you need a provider … that understands that there’s a lot to your health, in addition to trying to lose weight … So, I think somehow finding a way to address it without minimizing the struggle; that is, weight loss, and your whole health picture. Because I don’t think any of us don’t know that losing weight is good for us. I think it’s just that it can be complicated. (Participant #5, obesity group; white, Hispanic/Latino)

In addition to reports of dismissive and inadequate care for ongoing health concerns, women also described experiences with poorly coordinated care between providers. Participants reported that poorly coordinated care resulted in their health issues falling between the cracks of obstetrics and other care providers:

But I honestly, … I did not get the education from any of my physicians, and I saw a number of physicians over the years. Primary care, my OBGYN, etc. No one ever referred me to an endocrinologist. And that was something that I think in hindsight would have been really beneficial for me. Knowing that someone has polycystic ovarian syndrome … that has to do with insulin. More than likely, they will end up prediabetic or even diabetic. (Participant #unknown due to missing information on recording; prediabetes group)

This concern about poorly coordinated care between providers is especially germane when connected to a lack of handoff from OBGYN care to primary care, and when considering a lack of tailored health recommendations, as in the example of the following woman:

It feels like, after that six-week appointment, it’s … kind of hard to know how to process, … so I had *this,* now what’s that mean for me in five years, or 10 years, or down a long road? I have family history of diabetes. So, um, how worried should I be about this or things like that that I don’t think always get looped back around or sometimes discussed when they give you information that kind of is like, “Oh, checked the box. Gave her the … My Chart handout or the pamphlet or … mentioned exercising.” And though I never exercise… “See you next year.” (Participant #3; obesity group; white, non-Hispanic)

#### Suggestions for healthcare improvement

Women indicated their preferences for the optimal timing during the postpartum period to learn about their health risks and implications for their long-term health, as well as their preferred ways of learning this information. Due to the establishment of routines and a returned ability to mentally focus on health, participants suggested that the postpartum six-month or one-year mark would be an optimal time in which to revisit and prioritize, with their providers, health issues that require follow-up. Women agreed that the traditional six-week postpartum visit is not a conducive time to elaborate these details, as they are preoccupied with readjusting to life with a new baby

I think like six months after I have my baby, I’m a little more mentally stable. I don’t quite have the right words, but I’m not so emotional and more settled into, like, these are our routines. This is what we’re doing. Things have kind of gotten more back to normal. I can. I have the mental space to think about myself and these issues that we’re talking about. (Participant #5; pregnancy-complications group; white, non-Hispanic)

To fit the needs of different learning styles, women expressed the need for educational materials to be available in multiple formats (e.g., paper handouts, websites, videos, podcasts, doctor’s office informational group sessions). Overwhelmingly, however, they agreed that the information should also be available through a digital resource, such as their electronic health-record app, so that the information could be retrieved at their convenience:

Hand me everything digital because I can go back and look at it and reread it multiple times because I feel like it takes me a couple times to read it before I fully understand it. Especially with medical things. The terminology is hard, and so I read something, like, “I don’t even know what that’s saying” and so I need to look at it again and again, and so it’s nice if I have … it digitally, so that way, I can just keep going back to it and I won’t lose it. (Participant #2; pregnancy-complications group; white, non-Hispanic)

Women expressed a need for education that is “bite-sized information,” and that accounts for their role as busy moms. This abbreviated format could be incorporated into their daily routines, such as reviewing an educational video while washing dishes. Importantly, these types of resources could help to support an additional priority among participants-that this education be treated as an “ongoing conversation” designed to reinforce women’s learning over time. Women conveyed a desire for the avoidance of “one and done” education, sometimes referred to as providers “checking a box.” This need for brief educational resources that support ongoing learning exposes an additional appeal by the participants-the need for vetted and reputable sources of online information:

I spend a lot of time on social media. So, if … a doctor, or some credible source was giving out information, like, just like a helpful tip each day, or like a reel or something, that’s going to spark my interest. It’s going to be something I can go back to. And it’s something that I can share with other people, that I could do on my own, or with friends, and so I think social media would be a good platform, too. (Participant #1; pregnancy-complications group; white, non-Hispanic)

To summarize the participants’ suggestions for the improvement of women’s reproductive healthcare, women expressed a desire for 1) healthcare that is coordinated between providers (i.e., a clear hand-off between providers for women’s ongoing health conditions); 2) revisiting health risks between six-months to one-year postpartum; 3) educational materials that are available in multiple formats; and 4) education that can be treated as an ongoing conversation.

### Theme 3: Healthy Lifestyle-Change Barriers and Motivators

All women expressed interest in making healthy lifestyle changes, including engagement in formal programs, but they shared multiple barriers to healthy behavior change related to being “busy moms.” Women emphasized the need for social support and realistic solutions that accounted for the dynamics of motherhood and family life. Common motivators included the desire to maintain health for their families and to set a good example for their children. The research team sorted all codes into barriers and motivators categories, as listed in Table 2.

Barriers that have been discussed in previous sections of this paper include dismissive care from health providers and inadequate healthcare coordination between obstetrics and primary-care providers. In addition, multiple women highlighted a general lack of education as a barrier to engaging in healthy-lifestyle programs. One participant stated: “So, I think the biggest issue is a lack of education. It’s not a lack of doctors caring, but it’s a lack of time to teach, and follow-up regarding lifestyle changes.” (Participant #1; prediabetes group; white, non-Hispanic).

Participants shared unique insights on the difficulty of prioritizing one’s own health and wellness while planning for future pregnancies. Fatalism was noted as a barrier for one participant, who shared:

You have a baby, and then three years later you’re in good shape, and then you get pregnant again and you start all over again (laugh). So, part of me is like just whip them out now… be unhealthy for six years, and then, now I can actually get in shape and stay in shape. (Participant #4; pregnancy-complications group; white, non-Hispanic)

Self-sacrifice was a common barrier noted by multiple participants, with one noting, “I was focused on how to keep my baby safe, not [myself] 10 years from now… Like I don’t really care about that right now, I care about keeping my baby safe.” (Participant #5; pregnancy-complications group; white, non-Hispanic).

The postpartum period, in particular, was noted as a difficult time for women to receive health information and recommendations for wellness and lifestyle-change programs, as mothers are often struggling with the challenges of postpartum depression, mental ‘fuzziness,’ and feelings of being overwhelmed. One woman shared:

There’s already so much to do, as a mom, especially as a new mom, that, um…when you have a break…I feel like, for me, it was very much like, “I don’t want to do anything, I don’t wanna have to do one more thing.” Exercise was just one more thing … on my plate, and I couldn’t handle what was on it already. (Participant #4; pregnancy-complications group; white, non-Hispanic)

Cost was consistently mentioned as a key barrier to engagement in lifestyle-change programs. Participants noted the challenges of balancing the costs of parenting–including childcare–with the increased expense and time costs of buying and preparing healthy meals. One participant discussed her challenges as follows:

Meal prep and programs that deliver homemade meals to your door … that would be so helpful. But those cost, like, hundreds of dollars a month, and it’s just not something I would be able to afford. So, I kind of have to go cheap which doesn’t always mean a calorie deficit. It usually means I have to either do fast food or something that’s quick and easy. So yeah, there are childcare barriers, financial barriers, and definitely time. Time is a big one. (Participant #7; pregnancy-complications group; white, non-Hispanic)

While participants highlighted the barriers of childcare and family responsibilities in engaging in healthy lifestyle programs, multiple women expressed that their children also serve as a motivator to improve their health and wellness. One noted that she wants to improve her relationship with exercise to better her health for her family: “I don’t want to be in the background, not be able to do something with [my kids] because I didn’t put in the effort to keep myself healthy.” (Participant #3; obesity group; white, non-Hispanic).

## Discussion

This study provides important qualitative information to guide and improve the care of reproductive-aged women at risk for T2DM. Reproductive-aged women with cardiometabolic complications of pregnancy are at elevated risk for T2DM but are under-represented in lifestyle-change programs and prevention efforts. Participants highlighted a lack of continuity in care between their obstetric and primary-care providers, and voiced concerns regarding dismissive and/or perfunctory healthcare that minimized and poorly communicated their future health risks. Multiple barriers to T2DM-prevention efforts were highlighted, most notably lack of time, family and domestic labor commitments, and inadequate social and structured healthcare support during the postpartum period. Despite the concerns voiced by participants regarding their healthcare experiences, women reported that parenting and pregnancy were motivators for lifestyle changes and endorsed the need for improved efforts at T2DM-prevention programs for this patient population.

Fourth-trimester care is defined as postpartum maternal healthcare that occurs during the year following pregnancy. Although not routine in the United States, fourth-trimester care has been identified as an ideal opportunity for comprehensive cardiometabolic evaluation and handoff of care from obstetrics to primary and specialty providers.^25^ In addition to a six-fold increase in risk for developing T2DM, having a HDP increases a woman’s risk for cardiovascular mortality by 40%, contributing to the United States’ already significant burden of pregnancy-related morbidity or mortality.^26^ Such care could provide a means of supporting long-term health by formalizing the follow-up plan for pregnancy-associated cardiometabolic risk. Optimally, fourth-trimester care would be home-based and would leverage telehealth so as to minimize common barriers to postpartum care. Additionally, this care would include tailored guidance on postpartum health behaviors^25^ and could serve to educate women on the associations between pregnancy outcomes and their long-term health risks.

We learned from the participants of our study that their personal health-risk awareness was limited and superficial (e.g., being overweight might lead to other risks). Most women reported little understanding of their current health status and cardiometabolic risks, and rarely reflected awareness of their obstetric health complications and their impact on current health status (e.g., GDM increases their lifetime risk of developing T2DM). Additionally, participants reported that healthcare providers did not adequately follow up on and/or address their health risks, and they believed they lacked education and knowledge about their personal health risks. Similarly, a United Kingdom-based longitudinal qualitative study exploring women’s experiences of their postpartum care following HDP found women lacked tailored education from providers and educational materials about their health risk. Moreover, women were unaware of the implications of their personal risk profile for future pregnancies and for their long-term health.^27^ These findings were mirrored in a Norwegian-based qualitative study with women who had had GDM or preeclampsia, where participants described feelings of being left on their own to decipher the implications of their pregnancy complications and how to care for their health going forward.^28^ As a result, several participants reported mental-health struggles as they processed their diagnosis, need for self-advocacy, and complex motherhood transitions.

These findings are concerning as greater risk perception among women has been associated with engagement in a formal postpartum lifestyle-change program.^29^ Combined, these studies underscore the importance of healthcare providers effectively conveying long-term health risks associated with reproductive-aged women’s personal health profiles. In a report by Wilkinson et al., an Australian-based qualitative study with postpartum women who had had GDM or overweight/obesity, women said they desired a tailored and supportive lifestyle program offered by trusted professionals and women with lived experience that would account for the “new normal” of motherhood while also providing methods of accountability.^30^ The lack of time and energy that mothers face must be central to healthy behavior-change recommendations and to the design of lifestyle programs.^28^ These reported lifestyle-program priorities are similar to the priorities identified by women in the present study. Additionally, as in the present study, free or low-cost programs were identified as a priority of postpartum women.^31^ Having to pay for a healthy-lifestyle program was a barrier for this study’s participants, who cited a number of competing financial responsibilities.

This study has several strengths, including the participation of mothers with various cardiometabolic risk factors and a wide range of obstetric and medical diagnoses. Additionally, the study’s collaboration with the University of Utah’s CCET contributed to best-practices in qualitative and community-based research, including the use of the same interview guide and moderator for all three focus groups. Study limitations include the fact that the study population was majority white/non-Hispanic. Additionally, the experiences of women from a single intermountain region may be different from those living in different geographic regions. These women were savvy healthcare consumers, many of whom had participated in other studies with the CCET. Therefore, findings for women with less research experience may reveal even greater knowledge gaps and barriers to lifestyle-change program participation. Despite these limitations, this study provides helpful information about the knowledge and experiences of reproductive-aged women with cardiometabolic risk factors.

## Conclusions

Understanding reproductive-aged women’s views regarding their health status and risk for T2DM represents a key component in designing effective lifestyle programs. In our study, women demonstrated limited knowledge regarding the connection between pregnancy health and long-term health outcomes, such as potential diabetes-related health risks. Moreover, women identified numerous barriers to participation in lifestyle programs, yet reported being eager to receive further education. Overall, women were able to identify practical, real-world strategies supportive of their ability to participate in lifestyle programs. Nonetheless, further research is needed to understand diverse women’s experiences and identify potential barriers and facilitators.

## Figures and Tables

**Table 1:** Participant Demographics

Variable	Frequency (%) n = 20
Age, years Mean 34.2, Standard Deviation (6.46) / Range 26–48	
18–28	6 (30%)
29–39	10 (50%)
40+	4 (20%)
Race	
Asian	1 (5%)
Native Hawaiian or Pacific Islander	2 (10%)
White or European	15 (75%)
Multiracial	1 (5%)
Unreported	1 (5%)
Ethnicity	
Non-Hispanic/Latina	17 (85%)
Hispanic/Latina	3 (15%)
Partnership Status	
Married or living with a partner	15 (75%)
Divorced	2 (10%)
Separated	2 (10%)
Single	1 (5%)
Education	
Some college	2 (10%)
Associate’s degree	3 (15%)
Bachelor’s degree	14 (70%)
Master’s degree	1 (5%)
Setting	
Rural	1 (5%)
Suburban	13 (65%)
Urban	6 (30%)
Number of live births	
1	5 (25%)
2	6 (30%)
3	8 (40%)
4	1 (5%)
Number of children living in home	
1	5 (25%)
2	8 (40%)
3	6 (30%)
4	1 (5%)
Prior formal lifestyle program attendance	
Yes	8 (40%)
No	12 (60%)
Weight M 179.025, Standard Deviation (40.6740) / Range 125–280	

**Table 2:** Barriers and Motivators with Exemplar Quotes

Barrier Codes	Code Description	Exemplar Quote
Dismissive Care	Care that was described as dismissive of women’s healthcare needs and priorities	“Sometimes that’s a challenging area for me. Advocating for myself with my doctors or just pushing them to keep looking for why something weird is happening. You know, ‘I noticed this in my body’ or noticed that, and it’s just like, ‘oh, well keep an eye on it.’ And sometimes it just gets a little brushed off.” (Participant #3; obesity group; white, non-Hispanic)
Fatalism	Statements that reflect feelings of fatalism and futility as a barrier to lifestyle change	“You have a baby, and then three years later you’re in good shape, and then you get pregnant again and you start all over again (laugh). So, part of me is like, just whip them out now … be unhealthy for six years, and then, now I can actually get in shape and stay in shape … And so, part of that is the challenge of, ‘is it even worth it?’ Cause it just starts over.” (Participant #4; pregnancy-complications group; white, non-Hispanic)
Focused on the Present, Not the Future	Descriptions of women being focused on the present (e.g., pregnancy and neonatal outcomes) rather than long-term health	“I was focused on how to keep my baby safe, not necessarily 10 years from now … Like I don’t really care about that right now, I care about keeping my baby safe.” (Participant #5; pregnancy-complications group; white, non-Hispanic)
General Knowledge of Risk	Statements reflecting a generalized knowledge of risk that highlight a lack of in-depth knowledge related to women’s specific risks based on their risk profile	“I feel like I never got information about why [high blood pressure is] important or why that matters. Just that it’s scary and that it’s a problem but no information about why or how it could affect other pregnancies or just my life in general after.” (Participant #1; pregnancy-complications group; white, non-Hispanic)
Lack of Education	Statements describing how a lack of education from healthcare providers acted as a barrier to lifestyle change	“So, I think the biggest issue is a lack of education. It’s not a lack of doctors caring, but it’s a lack of time to teach, and follow up regarding lifestyle changes.” (Participant #1; prediabetes group; white, non-Hispanic)
Cost	Statements about cost and finances as barriers to engagement in lifestyle change	“Diet and exercise programs, normally those kinds of things are really expensive. As a new mom, and you know, a young married couple, we just don’t really have it in the budget for a long, big-commitment kind of program.” (Participant #1; prediabetes group; white, non-Hispanic)
Unappealing Format	Statements about program format types and forms of delivery as barriers to participation in lifestyle change	“If it’s only presented in one way, if it’s only an in-person class, I’m not gonna be able to attend a class necessarily, nor would I want to, as a more introverted person. So, I think that if there were not other methods available, that would prevent me from participating.” (Participant #3; prediabetes group; Hispanic/Latino)
Exercise	Statements about barriers to engaging in exercise	“I don’t want to go to a personal trainer because I feel like that is for somebody who loves to work out … and I absolutely hate it (laughs). And it is very hard for me … physically it’s hard. I have asthma that’s induced by exercise, and a lot of back problems.” (Participant #4; pregnancy-complications group; white, non-Hispanic)
Food	Statements about food and access to food as a barrier to lifestyle change	“For me, my husband, he loves eating the Pakistani food, which is a lot of oil, and spices. So overnight, like he says, like, ‘we’ll go to the fitness center together, but I want that diet. I can’t stop that.’ So, I for sure, I will make that stuff for him. But when it comes to me, I will say okay, I don’t have energy to make something for my own self. I’ll eat that. So that actually, you know, that environment or like… He has actually had a big impact on my diet, also. That’s, I think, a factor which actually prevents me to eat healthy.” (Participant #3; obesity group; Asian)
Healthcare Inadequacy	Inadequacies in healthcare described by participants	“With my pre-eclampsia, I didn’t have a six-week checkup. They just sent me home with a blood pressure cuff to monitor my blood pressures. So, I just relied on searching for answers myself. I really haven’t been able to get my blood pressures down after that, and that’s really all I’ve ever been told.” (Participant #6; pregnancy-complications group; Native Hawaiian/Other Pacific Islander)
Childcare	Lack of childcare described as a barrier to participation in lifestyle change	“Whether it’s incorporating my children in these habit changes, which is probably a good thing, or finding childcare to watch my kids while I go and try and make these lifestyle changes … um, that way, they’re not 100% in the way. There’s no in-between, at least for my lifestyle, so I would have to get childcare.” (Participant #7; pregnancy-complications group; white, non-Hispanic)
Pregnancy and Postpartum	Statements about pregnancy and the postpartum period as barriers to participation in lifestyle change	“When the pregnancy started, I don’t have energy to move an inch. So, that, is actually putting a big barrier for me to continue exercising or eating healthy because when you are pregnant, then you can’t do whatever you try.” (Participant #2; obesity group; Asian)
Delayed Results	Statements about how a lack of results can act as a barrier to participation in lifestyle change	“I think that something that might prevent me from participating in a formal program is if I didn’t see the benefit right away, you know?” (Participant #4; obesity group; white, non-Hispanic)
Lack of Social Support	Descriptions of a lack of social support as a barrier to participation in lifestyle change	“Sometimes people don’t have support from their spouse or their family to help them to get into the exercising or watching a baby and your other kids or anything so that you can take care of yourself.” (Participant #4; pregnancy-complications group; white, non-Hispanic)
Time	Statements about how a lack of time and scheduling conflicts can act as barriers to participation in lifestyle change	“Sometimes those classes are at a hard time for me. My kids are all in school now, but all these classes that I’ve seen are like … right when I need to be dropping off or picking up kids from school.” (Participant #4; pregnancy-complications group; white, non-Hispanic)
Lack of Healthcare Coordination	Descriptions of a lack of coordination between OB and other healthcare providers following pregnancy and the postpartum	“Even like a referral to say, ‘hey, after [delivery] you should follow up with your general doctor about all of these and that you have this, and you need to keep an eye on that for the future.’ I know that I did not ever … I did not receive that. and I don’t know that I’ve ever had any women that I’ve been in contact with, um, get that information after.” (Participant #2; pregnancy-complications group; white, non-Hispanic)
Weight Stigma	Statements about weight stigma	“I felt like they made it more about statistics, that you’re statistically overweight, and they just kind of glanced over me as a person.” (Participant #7; pregnancy-complications group; white, non-Hispanic)
Mental Health	Mental health described as a barrier to participation in lifestyle change	“The postpartum blues, getting yourself out of the house … at that point, like, for me I don’t care about myself. I care about this baby.” (Participant #4; pregnancy-complications group; white, non-Hispanic)
Motivator Codes	Code Description	Exemplar Quote
Accountability	Accountability described as a motivator to participation in lifestyle change	“I think what would encourage me to participate in one is actually knowing it’s a commitment, and not just kind of casual … knowing that it is a commitment, that I will be meeting with those same people … these people you see every Wednesday night, and they’re there to encourage you and to help you.” (Participant #1; prediabetes group; white, non-Hispanic)
Competition	Competition described as a motivator to participation in lifestyle change	“I’m a really competitive person. So, any type of competition and I go, ‘Yeah, I’ll do that.’ And so, just like a motivator for me is just competition. Which, I don’t know if that’s a good thing (laugh), but yeah.” (Participant #4; pregnancy-complications group; white, non-Hispanic)
Kids	Statements about how children and parenting can serve as a motivator to participation in lifestyle change	“I wanna be around for my kids and I want to teach my kids that this is a healthy attribute.” (Participant #1; pregnancy-complications group; white, non-Hispanic)
Knowledge	Knowledge described as a motivator to participation in lifestyle change	“I think knowledge is power for me. So, like, I know something is important, then I’m gonna want to do it. Especially if it affects my kids or myself … if we have adequate information and we know how to make changes that would actually help me, even if it’s smaller, then it would be easier to start.” (Participant #1; pregnancy-complications group; white, non-Hispanic)
Family/Anecdotal Experience	Family/social history described as a motivator to participation in lifestyle change	“I have families and friends that are going through this process of diabetes, and it is not … something that I want to go through. So, I really want to do something about it.” (Participant #4; prediabetes group; no race/ethnicity selected)
Monetary Incentives	Monetary incentives described as motivators to participation in lifestyle change	“I think [monetary] incentives is another motivation if we’re looking at time and priority. When we have incentives, then that kind of bumps up priority for certain people.” (Participant #5; prediabetes group; Native Hawaiian/Other Pacific Islander)
Positive Healthcare Interaction	Statements about positive healthcare interactions as a motivator to participation in lifestyle change	“I was always very grateful for the first doctor that spoke to me about it, really educated me about it. And, you know, not only sent me home with pamphlets, but took time in that meeting.” (Participant #1; prediabetes group; white, non-Hispanic)
Personalization	Importance of personalization of programming described as a motivator to participation in lifestyle change	“So, I think if it was something that was personalized, if someone sat down … and came up with a plan that was gonna work for me personally with my interest, with my preferences … and it was like doable, because we’re all busy, as mothers. I think if it’s something that feels … doable and is um, not that all-or-nothing thinking, and maybe more personalized to … the individuals’ preferences.” (Participant #5; pregnancy-complications group; white, non-Hispanic)

## Data Availability

The data sets for this study are not publicly available, as privacy of the study’s participants could be jeopardized. Data requests may be submitted to the corresponding author, and access to de-identified data may be permitted as determined by the University of Utah Institutional Review Board.

## Abbreviations

CCET: Community Collaboration & Engagement Team
DPP: National Diabetes Prevention Program
GDM: Gestational diabetes
HDP: Hypertensive disorder of pregnancy
T2DM: Type 2 Diabetes
